# Fractions and isolated compounds from *Oxyanthus speciosus* subsp. *stenocarpus* (Rubiaceae) have promising antimycobacterial and intracellular activity

**DOI:** 10.1186/s12906-019-2520-x

**Published:** 2019-05-22

**Authors:** Abimbola O. Aro, Jean P. Dzoyem, Maurice D. Awouafack, Mamoalosi A. Selepe, Jacobus N. Eloff, Lyndy J. McGaw

**Affiliations:** 1 Department of Paraclinical Sciences, Faculty of Veterinary Science, University of Pretoria, Private Bag X04, Onderstepoort, 0110 South Africa; 2 Department of Biochemistry, Faculty of Science, University of Dschang, P.O. Box 67, Dschang, Cameroon; 3 Laboratory of Natural Products Chemistry, Department of Chemistry, Faculty of Science, University of Dschang, P.OBox 67, Dschang, Cameroon; 4 Department of Chemistry, University of Pretoria, Pretoria, South Africa

**Keywords:** *Oxyanthus speciosus*, *Mycobacterium tuberculosis*, Rotundic acid, Lutein, Intracellular, Nitric oxide inhibition

## Abstract

**Background:**

Tuberculosis is a deadly disease caused by *Mycobacterium* species. The use of medicinal plants is an ancient global practice for the treatment and prevention of diverse ailments including tuberculosis. The aim of this study was to isolate and characterize antimycobacterial compounds by bioassay-guided fractionation of the acetone leaf extract of *Oxyanthus speciosus*.

**Methods:**

A two-fold serial microdilution method was used to determine the minimum inhibitory concentration (MIC) against mycobacteria. Cytotoxicity and nitric oxide inhibitory activity of the isolated compounds was determined to evaluate in vitro safety and potential anti-inflammatory activity. Intracellular efficacy of the crude extract against *Mycobacterium*-infected macrophages was also determined.

**Results:**

Two compounds were isolated and identified as lutein (1) and rotundic acid (2). These had good antimycobacterial activity against the four mycobacteria tested with MIC values ranging from 0.013 to 0.1 mg/mL. Rotundic acid had some cytotoxicity against C3A human liver cells. Lutein was not cytotoxic at the highest tested concentration (200 μg/mL) and inhibited nitric oxide production in RAW 264.7 macrophages by 94% at a concentration of 25 μg/mL. The acetone crude extract (120 μg/mL) of *O. speciosus* had intracellular antimycobacterial activity, reducing colony forming units by more than 90%, displaying bactericidal efficacy in a dose and time-dependent manner.

**Conclusion:**

This study provides good proof of the presence of synergism between different compounds in extracts and fractions. It is also the first report of the antimycobacterial activity of lutein and rotundic acid isolated from *Oxyanthus speciosus*. The promising activity of the crude extract of *O. speciosus* both in vitro and intracellularly in an in vitro macrophage model suggests its potential for development as an anti- tuberculosis (TB) herbal medicine.

## Background

Tuberculosis, caused by organisms belonging to the *Mycobacterium tuberculosis* complex, has re-emerged as a major disease of global importance University of Pretoria [1]. The innovation of tuberculin in 1890, Bacillus-Calmette Guerin (BCG) vaccine in 1908 and discoveries of antimycobacterial drugs in 1943 brought great hope for the eradication of this deadly disease until the pandemic of HIV/AIDS and upsurge of resistant strains (multi-drug, extensive-drug and total-drug resistant) ravaged humankind [2]. To effectively combat these drug resistant cases, new TB drugs with novel modes of action are desperately needed. After a long period of inactivity, there has been an increase in the number of new antimycobacterial drugs in the pipeline with the recent approval of bedaquiline and delamanid by the US food and drug administration (FDA) for the treatment of drug resistant TB [3]. However, these drugs are only used as a last resort due to their reported toxicity [4]. Therefore, novel, efficacious and safe anti-TB drugs that can shorten the duration of therapy, with fewer toxic effects to promote patient compliance is urgently needed. Drugs able to combat MDR, XDR and TDR-TB strains, active against latent TB and able to act in synergism with co-administered anti-TB drugs, are urgently required. Currently, there are increasing numbers of drug candidates in the optimization stage, preclinical development, phase II and phase III clinical trials. However, the low number of drug candidates in the phase I stage is worrisome in the eventuality of failure of advanced drug candidates [3]. The efforts of the TB Alliance are geared towards development of novel anti-TB drugs; preliminary screening of natural products for drug discovery is imperative to increase the number of drug candidates in the pipeline [5]. Furthermore, an interdisciplinary approach is needed for the discovery of new chemical molecules against both active and latent forms of TB [4].

Nitric oxide (NO) is a free radical involved in many biological processes with the ability to enhance bactericidal and tumoricidal activities of activated macrophages [6]. Excessive production of reactive oxygen species (ROS) generated can lead to inflammation by enhancing the release of cytokines and activation of enzymes such as lipoxygenases (LOXs) from inflammatory cells. LOX has been linked to several inflammatory diseases including TB [7]. The role played by the reactive oxygen and nitrogen intermediates during TB infection is not fully understood, though it is known that hydrogen peroxide produced by macrophages activated by cytokines has a mycobactericidal activity [8]. Hence, the overproduction of reactive oxygen and nitrogen intermediates could lead to inflammation [9]. An effective immune response to *M. tuberculosis* plays a crucial role in determining the establishment of disease [10]. However, the intricate interaction of *M. tuberculosis* with the immune system leads to the release of a vast array of cytokines by diverse cell types in response to infection [11]. Macrophages are target cells for mycobacterial infections and are solely responsible for intracellular killing of mycobacteria, and this is largely dependent on the cytokine environment [12].

It is well-established that natural products contribute significantly to the discovery and derivation of lead compounds and development of drugs that are introduced into the market. Interestingly, 65% of antibacterials approved for use between 1981 and 2010 were natural products or their derivatives, including currently employed TB drugs, for example rifampicin and the aminoglycosides [13]. Natural products found in higher plants are important sources of therapeutic and pharmacological agents, and different research groups across the globe are screening different plants for their biological activities [14, 15]. Large anti-TB bioprospecting screening programmes are currently in progress, and there is a renewed interest in natural sources for finding novel antimycobacterials [16]. Selecting plants that have shown excellent in vitro activity and subjecting them to further in vivo efficacy and toxicity studies may lead to development of effective and safer drugs against infectious diseases [17].

The Rubiaceae family contains 611 genera but only 48 genera have been studied and have good biological activity against different pathogenic bacterial strains [18]. There are 61 genera and 228 species native or naturalized in southern Africa [19]. The Rubiaceae family has played a significant role in drug discovery by providing molecules used as templates for the development of drugs [19]. Members of this family produce a large diversity of substances such as iridoids, indole alkaloids, anthraquinones, terpenoids (diterpenes and triterpenes), flavonoids and other phenolic derivatives [20]. This family is also characterised by the presence of other natural products such as iridoids (a group of monoterpenoids), methylxanthines (such as theobromine and theophylline) and anthranoids [21]. McGaw et al. [22] documented the anti-tubercular activity of close to 180 species of some medicinal plants in southern Africa used for TB-related complaints; however, only 4 species from the Rubiaceae family were reported.

A previous study reported the activity of acetone leaf extracts of 537 South African tree species against eight important microorganisms including *Mycobacterium smegmatis* with the aim of finding extracts with high activity and predicting which taxa could have a high priority for further investigation [23]. Six species from the Rubiaceae family had interesting activity against *M. smegmatis* and were investigated in more detail against pathogenic *Mycobacterium* species as well. Acetone leaf extracts from *Oxyanthus speciosus* had promising efficacy against non-pathogenic and pathogenic mycobacteria [24]. The aim of this study was to identify the antimycobacterial compounds by bioassay-guided fractionation of the acetone crude leaf extract of *Oxyanthus speciosus*. The cytotoxicity of fractions and isolated compounds as well as the nitric oxide inhibitory activity of the isolated compounds were determined while the intracellular activities of the crude extract were evaluated.

## Methods

### Plant material

The leaves of *Oxyanthus speciosus* were collected in February 2013 in the Lowveld Botanical Garden, Nelspruit, Mpumalanga, South Africa based on the labels of the trees. Plant material was identified by Magda Nel and a voucher specimen was deposited at the HGWJ Schweickerdt Herbarium of the University of Pretoria (PRU) under the voucher number PRU 120078. The leaves were air dried at room temperature, ground to a fine powder in a Macsalab mill (model 2000 LAB Eriez) and stored in closed glass containers in the dark until needed.

#### Extraction, fractionation and isolation

**Fig. 1:**
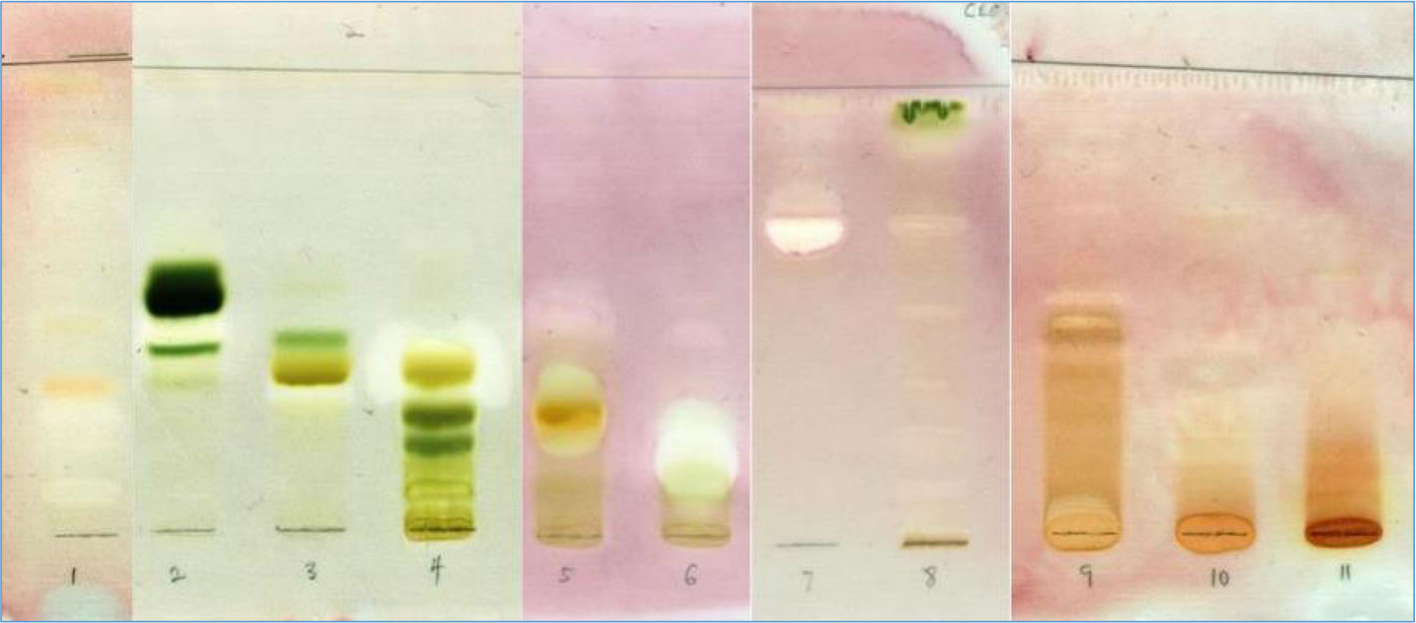
Bioautography of fractions against *M. smegmatis* showing clear bands of antimycobacterial activity in chromatograms developed in chloroform/ethyl acetate/formic acid (CEF)

The air-dried and powdered leaves of *O. speciosus* (170 g) were extracted in acetone (1 l) for 24 h (repeated thrice) to give a crude extract (40 g) after filtration and removal of the solvent at 40 °C using a rotary evaporator. Part of the crude extract (37 g) was subjected to silica gel column chromatography (CC) eluting with combinations of *n*-hexane (hex), ethyl acetate (EtOAc), and methanol (MeOH) in increasing polarity to afford 64 fractions of 500 mL each. Based on the similarity on the chromatograms from the thin layer chromatography (TLC) analyses, the collected samples were combined into eleven main fractions: F_1_ [hex/EtOAc (100:0 and 90:10), 750 mg], F_2_ [hex/EtOAc (90:10 and 80:20), 1.16 g], F_3_ [hex/EtOAc (80:20 and 70:30), 550 mg], F_4_ [hex/EtOAc (70:30), 1.14 g], F_5_ [hex/EtOAc (70:30 and 60:40), 610 mg], F_6_ [hex/EtOAc (50:50), 120 mg], F_7_ [hex/EtOAc (40:60), 160 mg], F_8_ [hex/EtOAc (20:80 and 0:100), 300 mg], F_9_ [EtOAc/MeOH (80:20), 30 mg], F_10_ [EtOAc/MeOH (50:50),10.49 g], and F_11_ [EtOAc/MeOH (30:70, 0:100), 11.03 g]. Based on the bioassay-guided activity, fractions F1 to F4 and F9, to F11 did not have as many and as active antimycobacterial compounds separated by TLC in bioautography (Fig. 1) as fractions F5, F6 to F8, and were not further investigated. Fractions F6 (80 mg) and F7 (120 mg) were individually subjected to repeated Sephadex LH-20 chromatography to afford compound **1** (6.4 mg). Fraction F8 (270 mg) was subjected to further silica gel column chromatography for purification using isocratic solvent system of *n*-hexane:acetone (7:3) to afford 48 sub-fractions of 50 mL each that were combined to main sub-fractions based on their TLC profiling. Sub-fractions F8 (12–13, 14–21, 22–37, and 38–48) yielded compound **2** (white powder, 50 mg). Fraction F5 (570 mg) was subjected to another silica gel column chromatography for purification using *n*-hexane: EtOAc in a gradient polarity eluent system. Due to the complex mixture of constituents and the small quantity of the material no compound was isolated.

#### General experimental procedures

The ^1^H and ^13^C NMR spectra were recorded with a Varian and Bruker (Avance III) spectrometer at 400 MHz for 1H and 100 MHz for ^13^C. Chemical shifts (*δ*) are quoted in parts per million (ppm) relative to the internal standard tetramethylsilane (TMS) or residual solvent peak (acetone-*d*_*6*:_ δ_H_ 2.05 and δ_C_ 29.84). The high-resolution mass spectra were recorded on a time-of-flight Waters Synapt high definition MS using electrospray ionization in the positive or negative mode. Column chromatography was performed on MN silica gel 60 (0.063–0.2 mm/70–230) mesh. Pre-coated plates of thin layer chromatography (TLC) silica gel 60 F254 (Merck, Germany) were used for monitoring fractions and spots were detected with UV light (254 and 365 nm) and then sprayed with vanillin-sulphuric acid spray reagent followed by heating at 110 °C for about three min until appropriate colour development.

Compound **1**: Orange powder, molecular formula: C_40_H_56_O_2_, ESIMS (+) m/z: 429 ([M-C_9_H_15_O]+); 1H NMR (100 MHz, methanol-d4) δH: 1.08 (H3–16), 1.08 (H3–17), 1.74 (H3–18), 1.97 (H3–19), 1.97 (H3–20), 1.01 (H3–16′), 0.86 (H3–17′), 1.63 (H3–18′), 1.92 (H3–19′), and 1.26 (H3–20′). 13C NMR (400 MHz, methanol-d4) δC: 65.9 (C-3), 65.0 (C-3′), 54.9 (C-6′), 48.4 (C-2), 42.5 (C-4), 44.6 (C-2′), 28.7 (C16), 30.2 (C-17), 21.6 (C-18), 12.7 (C-19), 12.7 (C-20), 24.3 (C-16′), 29.5 (C-17′), 22.8 (C-18′), 13.1 (C-19′), 12.8 (C-20′).

Compound **2**: White powder consisting of a mixture of closely related ursine-type triterpenes; ESIMS (+) m/z: 511.3397 [M + Na] + for C_30_H_48_O_5_Na, Calcd 511.3399; 1H NMR (400 MHz, acetone-d6) δH: 5.28 (1H, brt, 3.7 Hz, H-12), 3.56–3.62 (1H, m, H-3), 3.62 (1H, d, 10.5 Hz, H-23a), 3.32 (1H, d, 10.5 Hz, H-23b), 2.55 (1H, brs, H-18), 0.76 (3H, s, H3–24), 0.79 (3H, s, H3–25), 0.95 (3H, d, 6.8 Hz, H3–30), 0.97 (3H, s, H3–26), 1.20 (3H, s, H3–29), 1.35 (3H, s, H3–27);

#### GC-MS analysis of leaf extracts of *Oxyanthus speciosus*

The powdered plant material was extracted with acetone and analyzed using GC-MS LECO Pegasus 4D GC-TOFMS (LECO Africa (Pty) Ltd., South Africa). The data were obtained on an Elite-1(100% Dimethyl poly siloxane) GC column Rxi-5SilMS 30 m × 0.25 mm ID × 0.2 μm film thickness (Restek, Bellefonte, PA, USA). Spectroscopic detection by GC-MS involved an electron ionization system which utilized high-energy electrons (70 eV). Pure helium gas (Afrox, South Africa) was used as the carrier gas with a constant flow rate of 1 mL/min. Oven temperature was held for 3 min with 5 min solvent delay programmed at 40 °C and held isothermally at 300 °C for 5 min. An aliquot of 1 μL of acetone solution of the sample was injected in a spitless mode (spitless time 30s) and split ratio of 10:1 with the injector temperature at 250 °C and MS transfer temperature line set at 280 °C. Ion source temperature was maintained at 230 °C. A scan interval of 0.5 s and mass acquisition fragments ranging from 40 to 550 Da was maintained with data acquisition rate of 10 spectra/ s. The relative quantity of the compounds present in the extracts was expressed as a percentage based on the peak area produced in the chromatogram. Tentative identification of the bioactive constituents was based on the comparison of their retention time with those of standards samples and by matching the spectral fragmentation patterns against commercial library mass spectra.

### Antimycobacterial activity assay

#### Mycobacterial culture and inoculum preparation

Antimycobacterial activity was tested against four mycobacterial species including three non-pathogenic, fast-growing strains: *Mycobacterium smegmatis* (ATCC 1441) obtained from the American Type Culture Collection, *Mycobacterium aurum* (NCTC 10437) obtained from the National Collection of Type Cultures (UK Laboratory), *Mycobacterium fortuitum* (ATCC 6841) and one pathogenic strain *Mycobacterium tuberculosis* field strain (TB 8104) obtained from the Bacteriology Section, Agricultural Research Council-Onderstepoort Veterinary Institute, South Africa. Cultures were maintained as previously described [24].

### Cytotoxicity assay

The cytotoxicity of the crude plant extract, fractions and isolated compounds were tested against C3A human liver cells (purchased from ATCC, CRL-10741) using the 3-(4,5-dimethylthiazol)-2,5-diphenyl tetrazolium bromide (MTT) assay [25] with slight modifications [26].

### Nitric oxide (NO) inhibitory production

The NO production inhibitory activity of *Oxyanthus speciosus* crude extract and isolated compounds was evaluated in the LPS-activated mouse macrophage cell line RAW 264.7 as previously described [27]. Briefly, the percentage of nitric oxide released from the macrophages was assessed by determining the nitrite concentration in culture supernatant using Griess reagent. Post 24 h incubation, 100 μL of supernatant from each well of cell culture plates was transferred into 96-well microtitre plates and equal volume of Griess reagent was added. A microtiter plate reader (SpectaMax 190 Molecular devices) was used for reading the absorbance after 10 min at 550 nm. The concentrations of nitrite were calculated from regression analysis using serial dilutions of sodium nitrite as a standard. Validity of the assays was shown by using untreated cells as negative control, LPS-stimulated cells as positive control and additionally a cell group as reduction control group with LPS-stimulated cells, co-incubated together with quercetin used as an inhibitor of NO.

### Intracellular assay of the crude acetone extracts of *Oxyanthus speciosus*

The mouse macrophage cell line RAW 264.7 (ATCC TIB-71) was used to study the activity of samples against intracellular *M. fortuitum* [28, 29]. Cells were cultured in Dulbecco’s Modified Eagle Medium (D-MEM) supplemented with 10% FBS and 1% glutamine, at 37 °C and 5% CO_2_. Twenty mL of media (D-MEM) were dispensed into a 75- cm^3^ flask. Cells were detached from the flask by using a cell scraper, centrifuged at 1000 rpm for 5 min, suspended in 10 mL media and counted, after which the cell suspension was seeded in a 96-well microtitre plate at a density of 10^5^ cells/mL and incubated at 37 °C and 5% CO_2_ for 24 h. *M. fortuitum* grown in Middlebrook 7H10 broth was diluted with the cell culture medium (without antibiotics) to a final concentration of 10^5^ CFU/mL and added to each well to give a multiplicity of infection (MOI) of 3 and incubated for 4 to10 h at 37 °C in 5% CO_2_ for the cells to take up bacteria. Extracellular bacteria were removed by washing the plate with PBS and then 100 μL of drug-containing medium was added to each well followed by incubation under the same conditions as above. After 2, 4 and 6 days, cells from control and treated wells were lysed using 0.25% sodium dodecyl sulphate and sonicated at 1.5 W for 15 s. Then five dilutions (10^0^–10^5^) were prepared and 100 μL of each dilution were spread on Middlebrook 7H11 agar plates and incubated at 37 °C for 7 to 10 days until colonies were visible (4–6 days) and the number of CFU/mL was determined. A plant extract was considered bactericidal if it significantly reduced the colony forming units in the test samples compared with the control. Rifampicin serially diluted from 4 mg/mL (MIC = 50 μg/mL) was included as the control bactericidal compound.

### Statistical analysis

All experiments were conducted in triplicate and values expressed as mean ± standard deviation. Differences between values were assessed for significance using analysis of variance using Microsoft excel and results were compared using the Fisher’s least significant difference (LSD) at 5% significance level.

## Results

### Structure elucidation and GC-MS

**Fig. 2:**
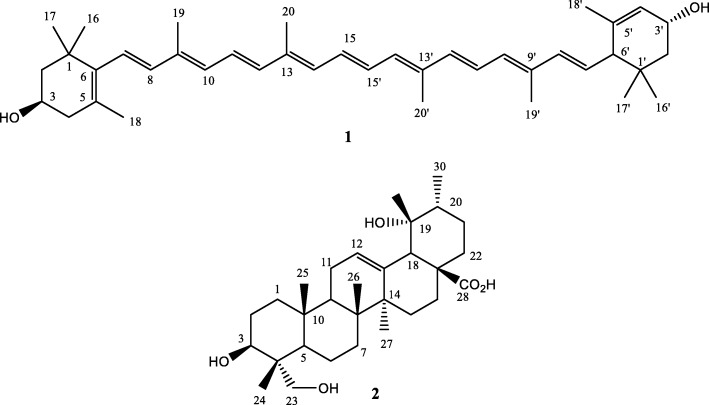
Chemical structures of lutein (1) and rotundic acid (2) isolated from *O. speciosus*

The acetone leaf crude extract of *O. speciosus* was subjected to bioassay-guided fractionation using open column chromatography and bioautography to determine the number of antimycobacterial compounds present [30] (Fig. 1). Briefly, the eluting solvent was removed from the chromatogram in a flow of air at room temperature, chromatograms were sprayed with a dense *M. smegmatis* culture, incubated overnight and sprayed with 0.2 mg/ml p-iodonitrotetrazolium violet to indicate Rf values of compounds that inhibited mycobacterial growth. The MIC of each fraction was also determined. Eleven main fractions were obtained. Two known compounds, lutein (1) [31–33] and rotundic acid (2) [34], were isolated (Fig. 2). The structures of these compounds were identified after analysis of their NMR data and by comparison with those reported in the literature. The presence of lutein (1) and rotundic acid (2) are reported from *Oxyanthus speciosus* for the first time to the best of our knowledge. The compositions of the crude extract constituents were established by GC-MS analyses (Table 1). The compounds present in the acetone extracts of *O. speciosus* were detected by gas chromatography (GC) and identified by mass spectrometry (MS). The GC-MS analyses revealed 17 compounds tentatively identified and confirmed with the library match of 70% similarity and above based on the peak area, retention time and molecular formula. To the best of our knowledge, no chemical or biological investigations have been carried out on this species as yet.

**Table 1:** Bioactive compounds detected in the acetone crude extract of *Oxyanthus speciosus*

S/N	Compound	RT (min)	Molecular formula	MW	% Peak Area
1	2-Methylthiophene	12.03	C_5_H_6_S	98	0.03
2	2-Methyl-penten-3-yne	1.33	C_6_H_8_	80	1.63
3	4-(Methylenecyclopropyl)-butyraldehyde or 4-(2-Methylenecyclopropyl) butanal	11.89	C_8_H_12_O	124	7.99
4	Methylcyclooctane	11.81	C_9_H_18_	126	0.34
5	*O*-Decylhydroxylamine	7.26	C_10_H_23_NO	173	3.92
6	4H-1,3,2-Dioxaborin, 6-ethenyl-2-ethyl-4-methyl-4-(2-methylpropyl)-	21.57	C_12_H_21_BO_2_	208	0.10
7	Phenyl 4-methoxybenzoate	19.62	C_14_H_12_O_3_	228	0.00007
8	3-Octadecyne	12.24	C_18_H_34_	250	0.87
9	2-Methylheptadecane	15.58	C_18_H_38_	254	1.94
10	Nonadecane	11.88	C_19_H_40_	268	7.99
11	Neophytadiene	24.67	C_20_H_38_	278	0.44
12	Phytol	14.75	C_20_H_40_O	296	0.73
13	1-Iodo-2-methylundecane	13.81	C_12_H_25_I	296	5.09
14	1,2-Benzenedicarboxylic acid, butyl 2-ethylhexyl ester	18.13	C_20_H_30_O_4_	334	0.05
15	Phthalic acid, heptyl pentyl ester	13.32	C_20_H_30_O_4_	334	0.02
16	Heptacosane	17.21	C_27_H_56_	380	0.87
17	2-(Cholest-5-en-3-yloxy) ethyl acetate	22.93	C_31_H_52_O_3_	472	0.92

*RT* Retention time, *MW* Molecular weight

### Antimycobacterial activity

The antimycobacterial activity of fractions and compounds from *Oxyanthus speciosus* was determined against three non-pathogenic and one pathogenic *Mycobacterium* species and the results are given as minimum inhibitory concentration (MIC) in mg/mL. An extract or fraction can be said to have a significant activity if the MIC value is 100 μg/mL or lower, moderate if 100 < MIC ≤625 and weak if MIC is > 625 μg/mL [35]. An isolated compound is said to possess significant antimicrobial activity if the MIC value is ≤10 μg/mL, moderate if 10 < MIC ≤100 μg/mL and weak if MIC is > 100 μg/mL [36]. The fractions had different degrees of activity against the four mycobacteria, ranging from excellent to weak with MIC values between 0.039 mg/mL and 2.5 mg/mL (Table 2). Fractions 9 and 10 had the best MIC value of 0.039 mg/mL while fractions 2, 3, 4, 5, 8 and 11 had moderate activity with MIC values ranging from 0.156 mg/mL to 0.625 mg/mL. Lutein and rotundic acid had moderate activity against the four tested mycobacteria with MIC values ranging from 12.5 to 100 μg/mL.

**Table 2:** Minimal inhibitory concentration (MIC in mg/mL) and total activity (TA) in L of extract, fractions and isolated compounds of *Oxyanthus speciosus*. (TA in L is calculated by dividing mass of fraction in mg (MF) by MIC in mg/mL and dividing the answer by 1000)

Samples	Yield (%)	MF (mg)	Microorganisms									
			*M. smegmatis*		*M. aurum*		*M. tuberculosis*		*M. fortuitum*		Average MIC	Average TA inL
			MIC	TA	MIC	TA	MIC	TA	MIC	TA		
Extract	2.99^a^	26,340	0.08^a^	329.3	0.06^a^	439.0	0.17^a^	154.9	0.078	337.7	0.097	315.2
F_1_	2.02	750	1.25	0.6	2.5	0.3	2.5	0.3	2.5	0.3	2.19	0.3
F_2_	3.15	1160	0.31	3.7	0.625	1.8	0.625	1.9	0.31	3.7	1.02	2.8
F_3_	1.48	550	0.15	3.7	0.31	1.8	0.31	1.8	0.31	1.8	0.27	2.2
F_4_	3.08	1140	0.15	7.6	0.31	3.7	0.31	3.7	0.156	7.3	0.27	5.6
F_5_	1.64	610	0.15	4.1	0.31	1.9	2.5	0.2	0.625	0.9	0.78	1.8
F_6_	0.32	120	1.25	0.09	2.5	0.048	0.625	0.2	1.25	0.1	1.25	0.1
F_7_	0.43	160	1.25	0.1	0.625	0.3	1.25	0.1	1.25	0.1	1.09	0.2
F_8_	0.81	300	0.15	2	0.625	0.5	2.5	0.1	1.25	0.2	1.13	0.7
F_9_	0.08	30	**0.039**	0.8	0.31	0.09	0.31	0.09	0.625	0.05	0.48	0.3
F_10_	28.35	10,490	**0.039**	**269**	0.156	**67.2**	0.31	33.8	0.625	16.8	0.28	**96.7**
F_11_	29.81	11,030	0.15	73.5	0.156	71	0.31	**35.6**	0.625	17.6	0.31	**33**
Rif	nd	nd	12.5	nd	1.56	nd	100	nd	nd	nd	38.02	nd
Lut	nd	6.4	0.05	na	0.013	na	0.025	na	0.025	na	0.028	na
RA	nd	50	0.1	na	0.1	na	0.1	na	0.1	na	0.1	na

*Ms M. smegmatis*, *Ma M. aurum*, *Mt M. tuberculosis, Mf M. fortuitum, F*_*1*_*-F*_*11*_ fractions, *nd* not determined, *na* not available, *RA* Rotundic acid, *Rif* Rifampicin, *Lut* Lutein

^a^MICs previously published by Aro et al. [24]

Values in bold indicates MIC and TA of fractions with good activites

Total activity (TA) of the fractions was calculated by dividing the total mass (mg) of the fraction by the MIC value (mg/mL) [35]. By dividing mass in mg by MIC in mg/mL the units of the result are in mL (mg/mg/ml = ml). Because the total activities of the fractions are so high, it is divided by 1000 and presented in L. The values obtained indicate the volume to which the active constituent present in the fraction can be diluted and still inhibit the growth of the tested organisms. F_10_ and F_11_ had the highest total activity against the tested organisms while F_6_ and F_7_ had the lowest total activity (Table 1). Also, TA calculation detects loss or gain in biological activity at each step of fractionation, by processes such as photo-oxidation or synergistic interaction between the plant fractions or compounds [35]. The crude acetone extract of *Oxyanthus speciosus* had TA values of 383and 0.063 L against *M. smegmatis*, *M. aurum* and *M. tuberculosis* (8104) respectively [24]. Total activity can also be used to select the most promising plant species to investigate [37]. In such a case the quantity in mg extracted (mg/mL) is divided by the MIC (mg/mL) providing a TA in mL/ mg. *Oxyanthus speciosus* subsp. *Stenocarpus* extracts were selected based on the TA*.* Total activity can also indicate if activity was lost or gained during the fractionation process [35]. The TA of the fractions ranged from 0.09 to 269 L. The acetone fraction had a total activity of 1771.1 L before it was further fractionated by column chromatography. The last two fractions had the highest total activity indicating that the active compounds were relatively non-polar. The total activity of the fractions against *M. smegmatis*, *M. aurum*, *M. tuberculosis* (8104) and *M. fortuitum* were 11.7, 13.5, 7.1. and 49.0 L respectively (Table 2).

### Cytotoxic activity and inhibition of NO production

Fractions F_9_ and F_10_ had some degree of toxicity against C3A human liver cells while other fractions had relatively moderate cytotoxicity against this cell line. Lutein was not cytotoxic even at the highest tested concentration (200 μg/mL) while rotundic acid was relatively cytotoxic to the tested cell line with an LC_50_ value of 33 μg/mL. Lutein also had low toxicity against RAW 264.7 macrophages, supporting the results found by Rafi and Shafaie [38]. The higher the selectivity index the higher the potential safety of the extract or compound when used in vivo. The selectivity index (SI) value obtained in this study for the fractions ranged from 0.02 to 3.27 with fraction F_8_ having the highest value against *M. smegmatis* (Table 3). Most of the fractions appeared to be more toxic to the human cells than to the microbes. Rotundic acid had a poor selectivity index of 0.33. The selectivity index (SI) of lutein ranged from 4 to > 16, indicating that the cytotoxic activities were higher to mycobacteria than to eukaryotic cells. Caamal-Fuentes et al. [39] stated that the therapeutic index of a drug should be 10 or higher. The two identified compounds isolated from the crude extract of *Oxyanthus speciosus*, lutein and rotundic acid, dose dependently inhibited NO production at concentrations of 3.12, 6.25, 12.5 and 25 μg/mL (Table 4). Lutein led to the highest percentage inhibition (94.99%) at a concentration of 25 μg/mL and a cell viability of 91.59%. The NO inhibitory activity expressed by lutein was therefore not due to a general metabolic toxin.

**Table 3:** LC_50_ (against C3A liver cells) and selectivity index (SI) values of extract, fractions and compounds of *Oxyanthus speciosus*

Samples	LC_50_ (μg/mL)	SI			
		*Ms*	*Ma*	*Mt*	*Mf*
Extract	383^a^	1.24^a^	2.46^a^	2.25^a^	4.91
F_1_	40	0.03	0.02	0.02	0.02
F_2_	34	0.11	0.05	0.05	0.11
F_3_	60	0.38	0.19	0.19	0.19
F_4_	190	1.22	0.61	0.61	1.22
F_5_	200	1.28	0.65	0.08	0.32
F_6_	70	0.06	0.03	0.11	nd
F_7_	156	0.12	0.25	0.12	0.12
F_8_	510	**3.27**	0.82	0.20	0.41
F_9_	40	1.03	0.13	0.13	0.06
F10	50	1.28	0.32	0.16	0.08
F11	80	0.51	0.51	0.26	0.13
Rif	> 200	nd	nd	nd	nd
Lut	> 200	> 4	> 16	> 8	> 8
RA	33	0.33	0.33	0.33	0.33
Dox	3.32	nd	nd	nd	nd

*RA* Rotundic acid, *Rif* Rifampicin, *Lut* Lutein, *Dox* Doxorubicin, *Ms M. smegmatis*, *Ma M. aurum*, *Mt M. tuberculosis, Mf M. fortuitum, F*_*1*_*-F*_*11*_ fractions, *nd* not determined

^a^previously published by Aro et al. [24]

Values in bold indicates fractions with the highest selectivity indices

**Table 4:** Inhibitory activities of compounds on NO production in LPS-activated RAW 264.7 macrophages

Samples	Concentration (μg/mL)	NO production (μM)	% NO inhibition	% cell viability
Lutein	25	0.14 ± 0.02	94.99	91.59
	12.5	0.44 ± 0.15	83.85	92.59
	6.25	1.37 ± 0.07	49.46	86.84
	3.12	1.98 ± 0.08	27.17	74.20
Rotundic acid	25	0.48 ± 0.18	82.39	87.48
	12.5	1.19 ± 0.16	56.24	86.11
	6.25	2.01 ± 0.16	26.20	78.51
	3.12	2.39 ± 0.09	12.16	73.73
Quercetin	25	0.35 ± 0.10	99.35	57.60
	12.5	0.30 ± 0.05	106.61	79.23
	6.25	0.69 ± 0.08	110.49	105.03
	3.12	2.50 ± 0.48	94.50	101.23

Values are expressed as mean ± SD

### Intracellular assay

**Fig. 3:**
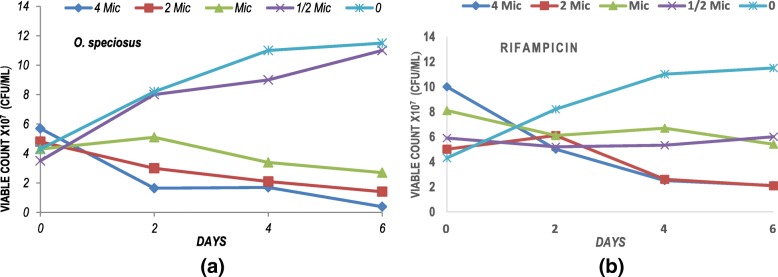
RAW 264.7 cells infected with *Mycobacterium fortuitum* with a MOI 1:5 (cells:bacilli) were exposed to several concentrations of extract and rifampicin (0.5X, 1X, 2X and 4X MICs, 0 = untreated control) in triplicate. After 2, 4 and 6 days post infection, cells were lysed and plated on 7H10 agar to determine CFU/mL. Plots detail the bactericidal activity of extract of *O. speciosus* (**a**) and rifampicin (**b**) used as the reference positive control antibiotic. Values represent means ± SD

The intracellular antimycobacterial activities of *O. speciosus* extract and the anti-TB drug rifampicin against *M. fortuitum* were assessed using RAW 264.7 macrophages. Mouse macrophages were infected with *M. fortuitum* with a multiplicity of infection (MOI) of five mycobacteria per cell. Infection of macrophages at a low MOI leads to phagosomal maturation thereby resulting in inhibition of *M. tuberculosis* growth in the macrophage [40]. The acetone extract of *O. speciosus* were not cytotoxic to RAW 264.7 macrophages even at the highest tested concentration (1 mg/mL) [27]. The extract significantly decreased the number of intracellular mycobacteria at 0.5X, 1X, 2X and 4X the MIC value of 120 μg/mL. The plate was washed with PBS before lysing the cells to avoid false positive results. *M. fortuitum* was effectively phagocytized after the 4 h incubation period. One unique characteristic of *Mycobacterium* species is the ability to grow in both intra- and extracellular environments, therefore an ideal antimycobacterial agent should be active in both locales [41]. On day 6 post-infection, the intracellular antimycobacterial activity of the acetone crude extract of *O. speciosus* at 1X to 4X MIC was superior to that of rifampicin, showing more than 90% reduction in colony forming units. The bactericidal activity observed was both dose and time-dependent. Moderate inhibitory effect was observed for the extracts of *O. speciosus* at 0.5X MIC inhibiting less than 50% growth of intracellular bacteria during 6 days of drug exposure (Fig. 3a) compared to that of rifampicin (Fig. 3b) at the same concentration, inhibiting more than 50% growth of the intracellular mycobacteria.

## Discussion

Discovery of potential lead compounds and their advancement towards drug development involves extraction of the crude extract from source, conducting bioassay-guided fractionation and most importantly the purification process to yield a single bioactive compound [42]. Crude natural product extracts are complex mixtures of perhaps hundreds of different compounds working together in synergy when the extract is administered as a whole. With the exception of *M. smegmatis* (that had an increase of 11%) there were substantial losses in the combined total activity of the fractions compared to the crude extract based on the fractionation assay. These losses varied from 86% (*M. fortuitum*)*,* 66% (*M. aurum*) to 50% for *M. tuberculosis*. The losses may be explained by inactivation of compounds during the isolation process or alternatively by the loss of synergism between compounds that were separated during the isolation. Therefore, the combined action of two or more substances can result in a biological effect higher than any single one’s. Hence, more thorough studies are necessary to find which substances should be mixed in order to attain the desired antimycobacterial activity because the two-fold serial dilution method does not provide accurate results in doubling or halving of the TA which may not be significant. The losses found here are much larger than explicable by inaccurate measurements. It is interesting that there were also large differences in losses between the different pathogens. The results obtained from this study is similar to the study conducted by Ntutela et al., [43] where loss of antimycobacterial activities after fractionation and effect of synergism between fractions were recorded.

Two compounds 2-(2-hydroxy)-ethanol-β-D-glucopyranoside and a cyanogenic glycoside, halocalin, were isolated from *Oxyanthus speciosus* subsp. *gerrardii* and *Oxyanthus speciosus* subsp. *speciosus* respectively [44]. Two compounds have been reported to be isolated from *Psychotria capensis* (Rubiaceae) and have been identified as β-sitosterol and a carotenoid derivative, lutein [45]. Plant carotenoids are naturally occurring fat-soluble pigments that give bright coloration to plants and have pharmacological properties such as strong antioxidant activity; they are also used for the relief of some chronic diseases like cardiovascular disease, osteoporosis and cancer [46]. Some carotenoids such as β-carotene, lutein and lycopene can also offer protection against some inflammatory responses [47]. Lutein has been isolated from *Tagetes erecta* and the plant is used for the treatment of cough and dysentery amongst others [48]. Some of the triterpenes isolated from the Rubiaceae family include oleanolic acid, ursolic acid, lupeol, betulinic acid, rotundic acid, barbinervic acid and luculiaoic acid (A) [20]. Rotundic acid was isolated from the fruit of *Ilex rotunda* [34]. This ursene-type triterpene was also isolated from the leaves of *Guettarda pohliana* belonging to the Cinchonoideae subfamily of the Rubiaceae family [49]. Previous reports have suggested that lutein is able to enhance in vitro and in vivo inflammatory responses by suppressing NF-ĸB activation [50, 51]. Therefore, it can be said that lutein plays a significant role in modulating inflammatory processes by regulating cellular redox potential [52]. The results obtained from this study suggest that the antimycobacterial activity exhibited by lutein could occur by inhibiting the production of inflammatory mediators in vivo responsible for the pathogenesis of chronic inflammatory disease such as TB. The ability of the extract to inhibit the replication of mycobacteria intracellularly revealed a noteworthy result. The low MIC value and dose-dependent bactericidal activity observed in the macrophages suggests effective intracellular penetration of the crude extract of *O. speciosus*. Although this observed inhibitory activity on intracellular mycobacteria was at a higher concentration (≥ 2X MIC), the LC_50_ of the crude extracts on the host cells was still higher (393 μg/mL) than this value. Therefore, the host cells would not be too adversely affected at concentrations that are effective against intracellular mycobacteria. The result obtained from this study is similar to the study conducted by Gupta et al., [53] where the acetone crude extract of *Alpinia galanga* was able to inhibit the replication of *Mycobaterium tuberculosis* intracellularly. The intracellular killing potential of the acetone extract of *O. speciosus* to exert its mode of action is of great interest.

## Conclusions

It can be concluded that lutein is one of the active compounds responsible for the antimycobacterial activity in the crude extract of *Oxyanthus speciosus*. In addition to the promising in vitro antimycobacterial activity and low cytotoxicity to C3A human cells, lutein also had good anti-inflammatory activity. The promising activity of the crude extract of *O. speciosus* both in vitro and intracellularly in macrophages suggests its potential for use as an anti-TB herbal medicine. However, further studies are necessary to investigate its mechanism of action, bioavailability and in vivo effects. This study also contributes to the validation of the use of non-pathogenic mycobacteria as a model for intracellular study.

## Abbreviations

BCG: Bacillus-Calmette Guerin
CC: Silica gel column chromatography
FDA: Food and Drug Administration
hex: N-hexane
LOX: Lipoxygenases
MDR: Multiple Drug Resistance
MIC: Minimum Inhibitory Concentration
NO: Nitric Oxide
PRU: University of Pretoria
RO: Reactive Oxygen Species
TA: Total Activity TA – Total Activity
TDR: Totally Drug Resistant
XDR: Extensively Drug Resistant
